# Hot Milk a Stimulant

**Published:** 1891-09

**Authors:** 


### HOT MILK A STIMULANT.

Milk heated to much above 100 degrees, Fahrenheit, loses for a time a degree of its sweetness and density. No one who, fatigued by over-exertion of body or mind, has ever experienced the reviving influence of a tumbler of this beverage, heated as hot as it can be sipped, will willingly forego a resort to it because of its being rendered somewhat less acceptable to the palate. The promptness with which its cordial influence is felt, is indeed surprising. Some portion of it seems to be digested and appropriated almost immediately, and many who now fancy they need alcoholic stimulants when exhausted by fatigue, will find in this simple draught an equivalent that will be abundantly satisfying and far more enduring in its effects.

There is many an ignorant, overworked woman who fancies she could not keep up without her beer ; she mistakes its momentary exhilaration for strength, and applies the whip instead of nourishment to her poor, exhausted frame. Any honest, intelligent physician will tell her that there is more real strength and nourishment in a slice of bread than in a quart of beer ; but if she loves stimulants it would be a very useless piece of information. It is claimed that some of the lady clerks in our own city, and those, too, who are employed in respectable business houses, are in the habit of ordering ale or beer in the restaurants. They probably claim that they are "tired," and no one who sees their faithful devotion to customers all day will doubt their assertions. But they should not mistake beer for a blessing, or stimulus for strength. A careful examination of statistics will prove that men and women who do not drink can endure more hardships and more work, and live longer than those less temperate.—*Ex.*

